# Identifying causes of loss to follow up in newly diagnosed HIV-infected patients

**DOI:** 10.1186/1758-2652-13-S4-P124

**Published:** 2010-11-08

**Authors:** V Fridman, NS Bello, MB Lasala

**Affiliations:** 1 Hospital de Clinicas, Infectious Diseases, Buenos Aires, Argentina; 2 Hospital de Clinicas Jose de San Martín, Infectious Diseases, Buenos Aires, Argentina

## Purpose of the study

The aim of the study was to evaluate the cause of the lost to follow-up in the newly diagnosed HIV positive patients, after the first visit in the HIV clinic.

## Methods

We retrospectively reviewed the clinical charts of the adult patients (P) who consulted for the first time in the Infectious Disease Clinic of the university associated hospital at Buenos Aires, Argentina, between June 1st 2005 and May 31st 2008. We identified between the P who were newly diagnosed with HIV infection, those who attended the clinic only once; we considered lose to follow up those P who never come back in a year time. We exclude those P who attended other clinic before coming to ours. We contacted via telephone those P who had only one visit and never came back, to know the reason why they never came back to the clinic; we compared the age, gender, and socioeconomic characteristics between the group of patient who continued medical care (those who are currently attending our clinic and were diagnosed in the same period) and those who lost to follow up. We considered the first visit, the one in which the P is informed about his HIV seropositive condition.

## Summary of results

We included 227 adult P who consulted the clinic for the first time. We identified 123 P (54%) who never came back to consultation after the first visit. The main cause of lost to follow up was because the patient felt good enough and thought they do not need medical attention (31/123); 25/123 P gave us a wrong number so we could not contact them. Most of the P were under no medical attention at the time we called them (61/123). We compare those P with 104 P who continued attending our clinic; the group of P who continued under medical care were older (median age 40.4 vs 34.2), patient with 40 years or more were 50/104 in the first group and 24/123 in those lost to follow up (p=0.0000048 OR 3.82 CI 2.04-7.20), and were in most cases women (34/104 vs 30/123, p=0.16 OR1.51 (0.81-2.80). P who continue follow up were working in most cases (95/104 vs 45/123, p=0.0000 OR 18.3 CI 8.00-43.11), live with family/partner (94/104 vs. 77/123 p=0.01 OR 0.67) and got better scholarship degree (university studies 21/104 vs 7/123 p=0.000 OR 4.19 CI 1.60-11.43) than P that did not continue follow up.

## Conclusions

We must reinforce the medical care need in recently diagnosed HIV patients during the first visit to avoid the loss to follow up, especially in those patients who are younger and socially excluded.

